# IgA, albumin, and eosinopenia as early indicators of cytomegalovirus infection in patients with acute ulcerative colitis

**DOI:** 10.1186/s12876-020-01434-5

**Published:** 2020-09-05

**Authors:** Hong Yang, Kaichun Wu, Hongjie Zhang, Qin Owyang, Yinglei Miao, Fang Gu, Naizhong Hu, Kaifang Zou, Jianqiu Sheng, Jin Li, Ping Zheng, Yulan Liu, Junxia Li, Xiaodi Wang, Yongdong Wu, Yaozong Yuan, Chunxiao Chen, Yanhua Pang, Meihua Cui, Jiaming Qian

**Affiliations:** 1 Department of Gastroenterology, Peking Union Medical College Hospital, Chinese Academy of Medical Science & Peking Union Medical College, Beijing, 100730 P.R. China; 2 Department of Gastroenterology, Xijin Hospital, The Fourth Military Medical University, Xi’an, China; 3 Department of Gastroenterology, Jiangsu Province Hospital, Nanjing, China; 4 Department of Gastroenterology, West China Hospital, Sichuan University, Chengdu, China; 5 Department of Gastroenterology, First Affiliated Hospital of Kunming Medical University, Kunming, China; 6 Department of Gastroenterology, Peking University Third Hospital, Beijing, China; 7 Department of Gastroenterology, First Affiliated Hospital of Anhui Medical University, Hefei, China; 8 Department of Gastroenterology, Tongji Hospital of Tongji Medical College of Huazhong University of Science and Technology, Wuhan, China; 9 Department of Gastroenterology, General Hospital of Beijing Military Region, Beijing, China; 10 Department of Gastroenterology, Zhongnan Hospital of Wuhan University, Wuhan, China; 11 Department of Gastroenterology, Shanghai General Hospital, Shanghai, China; 12 Department of Gastroenterology, Peking University People’s Hospital, Beijing, China; 13 Department of Gastroenterology, Peking University First Hospital, Beijing, China; 14 Department of Gastroenterology, China-Japan Friendship Hospital, Beijing, China; 15 Department of Gastroenterology, Beijing Friendship Hospital of Capital Medical University, Beijing, China; 16 Department of Gastroenterology, Ruijin Hospital of Shanghai Jiaotong University School of Medicine, Shanghai, China; 17 Department of Gastroenterology, First Affiliated Hospital of Zhejiang University, Beijing, China; 18 Department of Gastroenterology, Beijing Chao-Yang Hospital, Beijing, China; 19 Department of Gastroenterology, Aerospace Central Hospital, Beijing, China; 20 Department of Gastroenterology, Peking Union Medical College Hospital, Peking Union Medical College, Chinese Academy of Medical Sciences, Beijing, 100730 P.R. China

**Keywords:** Ulcerative colitis, Cytomegalovirus, Glucocorticoid, Immunosuppressive agents, eosinophils

## Abstract

**Background:**

Cytomegalovirus (CMV) infection can significantly complicate and worsen the condition of acute severe ulcerative colitis (UC) patients. We aimed to explore the predictive risk factors to prevent and identify CMV infection at an early stage in acute UC patients.

**Methods:**

A total of 115 moderate-to-severe active UC patients from 17 hospitals throughout China were enrolled. Active CMV infection was diagnosed by one of the following: CMV pp65 antigens, CMV IgM antibodies or CMV DNA. We identified the independent risk factors by multivariate analyses.

**Results:**

A total of 64 of 115 active UC patients had active CMV infection. Compared to the non-CMV-infected patients, the CMV-infected patients had a tendency to be male and to exhibit abdominal pain; fever; oral ulcers; eosinopenia; low albumin, immunoglobulin (Ig) A, IgM, and IgG levels; increased high-sensitivity C-reactive protein (hsCRP) levels; hyponatremia; pancolonic lesions; initial onset type; severe activity; and glucocorticoid (high-dose) and immunosuppressive agent use (*P* < 0.05). In further multivariate analyses, the use of high-dose glucocorticoids (OR 13.55, 95% CI 2.49–73.61, *P* < 0.01) and immunosuppressive agents (OR 11.23, 95% CI 1.05–119.99, *P* = 0.04) were independent risk factors for CMV infection. A decrease eosinophil and albumin levels were risk factors for CMV infection. With every 0.1*10^9/L decrease in the peripheral blood eosinophil level or 1 g/L decrease in the serum albumin level, the risk for CMV infection in UC patients increased by 5.21-fold (1/0.192) or 1.19-fold (1/0.839), respectively.

**Conclusions:**

High-dose glucocorticoid and immunosuppressive agent treatment significantly increase the risk of CMV infection, and correcting eosinopenia and low albumin levels may help prevent CMV infection in UC patients.

## Instruction

Ulcerative colitis (UC), a type of nonspecific inflammation of the colon, is characterized by a chronic disease course and delayed healing. The morbidity and colectomy rates of UC patients have significantly decreased with the development and application of therapeutic drugs such as glucocorticoids and immunosuppressive as well as biological agents. However, the use of these therapeutic drugs alone or in combination may increase the risk of opportunistic infections. In particular, it has been shown that cytomegalovirus (CMV) infection can complicate the condition or even increase the death rate of UC patients [1]. CMV, belonging to the herpesviridae family, remains latent after the initial infection and persists throughout the lifetime of the host. Indeed, patients with inflammatory bowel disease (IBD) are often immunosuppressed and severely malnourished, which may lead to a significantly increased risk of CMV reactivation [2]. Twenty-one to 34 % of acute and severe UC patients are reportedly CMV-positive, and the active CMV infection rate among those who receive urgent colectomy for UC is 10–33.3% [3–7].

One study [8] showed that female gender, pancolic inflammatory disease and active inflammation upon histological examination were independent risk factors for CMV reactivation. However, few studies have focused on blood markers for predicting the risk of CMV infection in IBD patients. Such markers will be helpful for clinicians to monitor and improve the disease prognosis as well as to initiate early intervention strategies for preventing infection. The goal of this study was to fully understand CMV infection in Chinese patients with active UC and to identify the risk factors for CMV infection in IBD patients with different demographic characteristics.

## Patients and methods

### Patients

A total of 115 patients from 17 hospitals with moderate-to-severe active UC and CMV infection were enrolled in this study from 2009 to 2017. The diagnostic criteria, disease activity, and lesion extent were based on the European Crohn’s and Colitis Organization (ECCO) guidelines [9] and the Consensus on the Diagnosis and Treatment of Inflammatory Bowel Disease (Beijing, 2018) [10]. All patients were diagnosed by clinical, endoscopic and histological studies. Disease severity was determined clinically by the Truelove and Witts’ score and endoscopically by the Mayo endoscopic score as previously described [11]. CMV infection was confirmed by blood pp65 antigens, CMV IgM antibodies and CMV qPCR. This study was approved by the Institutional Review Board of Peking Union Medical College Hospital.

### Peripheral blood CMV detection

The diagnostic standard for active CMV infection was a positive result using one of the following methods: HCMV pp65 antigen (CMV pp65), CMV IgM, and CMV DNA quantitative polymerase chain reaction (qPCR) detection. HCMV pp65 antigen detection was performed using immunofluorescence staining (HCMV Brite kit, IQ, the Netherlands). Detection of peripheral blood IgM antibodies was achieved using an enzyme-linked immunosorbent assay (ELISA) (Captia Cytomegalovirus IgM, Trinity Biotech Plc, Ireland). Quantitation of HCMV DNA was based on real-time qPCR (an HCMV nucleic acid quantitative assay kit, DAAN Gene Co., Ltd. of Sun Yat-sen University, China). The cut-off value of CMV-DNA in diagnosing CMV infection was 500 copies/ml. CMV colitis was defined as the identification of characteristic intranuclear or intracellular inclusion bodies on H&E sections and/or identification of CMV-specific antigen by IHC from colonoscopy biopsy samples.

### Steroid and immunosuppressive agents

The definitions of steroid and immunosuppressive agent application are described below [12]. The large steroid dose was defined as oral prednisone at ≥40 mg per day (qd). The moderate dose was defined as oral prednisone at ≥20 mg but < 40 mg qd for at least two months. The small dose was defined as oral prednisone at < 20 mg qd or oral prednisone at ≥20 mg qd for less than two months. The application of immunosuppressive agents was defined as the use of azathioprine, cyclosporine, or thalidomide in the past month.

### Statistical methods

The clinical symptoms, Montreal classification, complications, and laboratory indicators of confirmed UC patients with or without CMV infection were retrospectively compared and analyzed. Statistical analyses were performed using SPSS 19.0 software for univariate and multivariate analyses. In univariate analyses, categorical variables were analyzed using the chi-square or Fisher’s exact test. Continuous variables following a normal distribution are presented as the mean (SD) and were analyzed by t test; otherwise, they are presented as median and interquartile ranges and were analyzed by the Mann-Whitney U test. Indicators at *P* < 0.05 were included in binary logistic regression for multivariate analyses. P < 0.05 indicated statistical significance.

## Results

### Baseline characteristics

Of 115 patients with confirmed active UC, 64 had active CMV infection. CMV-DNA was higher than 500 copies/ml in 56 cases (87.5%), CMV-PP65 was positive in 24 cases (37.5%) and CMV-IgM was positive in 13 cases (20.3%). CMV colitis was diagnosed in 39 cases (60.1%) based on identification of characteristic intranuclear or intracellular inclusion bodies on H&E sections and/or identification of CMV-specific antigen by IHC.

In the active CMV infection group, the male/female ratio was 41:23, and the mean age was 47.63 ± 13.71 years. For the non-CMV infection group, the male/female ratio was 21:30, with a mean age of 44.69 ± 15.28 years. Between the two groups, the male/female ratio was significantly different: more males were in the CMV infection group, while more females were in the non-CMV infection group (*P* < 0.05). Conversely, age at disease onset (43.91 ± 13.17 vs. 38.78 ± 14.57) and current admission (47.63 ± 13.71 vs. 44.69 ± 15.28) did not differ significantly between these two groups. The two groups did not present significant differences in the history of diabetes mellitus, appendectomy, smoking, or drinking or in the familiar history of IBD. The specific data are summarized in Table 1.

**Table 1:** Baseline characteristics of patients in the CMV infection and non-CMV infection groups

Indicator	CMV infection (*n* = 64)	Non-CMV infection (*n* = 51)	*P*
Gender ^a^			0.014
Male	41 (64.1%)	21 (41.2%)	
Female	23 (35.9%)	30 (58.8%)	
Age at disease onset^b^	43.91 ± 13.17	38.78 ± 14.57	0.051
Age at current admission^b^	47.63 ± 13.71	44.69 ± 15.28	0.280
Smoking ^a^			0.079
Yes	22 (34.4%)	10 (19.6%)	
No	42 (65.6%)	41 (80.4%)	
Drinking ^a^			0.734
Yes	14 (22.2%)	10 (19.6%)	
No	49 (77.8%)	41 (80.4%)	
IBD family history^a^			0.380
Yes	4 (6.3%)	1 (2.0%)	
No	60 (93.7%)	50 (98.0%)	
Appendectomy history^a^			0.502
Yes	2 (3.1%)	0	
No	62 (96.9%)	51 (100%)	

^a^Chi-square test or Fisher’s exact test was used. ^b^ data are expressed as the mean ± SD and were determined by a t test. In the CMV infection group, the patient numbers for drinking do not add up to the total due to missing data

### Comparison and analyses of clinical manifestations, complications, and laboratory tests between the active UC with and without active CMV infection groups

As shown in Table 2, the incidence rates of abdominal pain (78.1% vs. 60.8%, *P* = 0.043) and fever (56.3% vs. 29.4%, *P* = 0.005) in patients with active CMV infection were higher than those in the noninfection group. Regarding extra-intestinal manifestations, he incidence of oral ulcers in the infection group was higher than that in the noninfection group (21.9% vs. 2.0%, *P* = 0.002). Complications between the two groups were not significantly different.

**Table 2:** Analysis of clinical characteristics between the CMV infection and non-CMV infection groups

Variable	CMV infection (n = 64)	Non-CMV infection (n = 51)	P^a^
**Clinical manifestation**			
Abdominal pain			0.043
Yes	50 (78.1%)	31 (60.8%)	
No	14 (21.9%)	20 (39.2%)	
Stool frequency	9.7 ± 6.4	8.0 ± 4.7	0.127
Hematochezia			0.746
Yes	59 (92.2%)	45 (90.0%)	
No	5 (7.8%)	5 (10.0%)	
Fever			0.005
Yes	36 (56.3%)	15 (29.4%)	
No	28 (43.8%)	36 (70.6%)	
**Parenteral presentations**			
Oral ulcer			0.002
Yes	14 (21.9%)	1 (2.0%)	
No	50 (78.1%)	50 (98.0%)	
Vulvar ulcer			1.000
Yes	1 (1.6%)	0	
No	63 (98.4%)	51 (100%)	
Arthritis			0.115
Yes	6 (9.4%)	10 (19.6%)	
No	58 (90.6%)	41 (80.4%)	
Gallstone			0.628
Yes	3 (4.7%)	1 (2.0%)	
No	61 (95.3%)	50 (98.0%)	
Eye lesion			0.584
Yes	1 (1.6%)	2 (3.9%)	
No	63 (98.4%)	49 (96.1%)	
Thromboembolism			0.321
Yes	1 (1.6%)	3 (5.9%)	
No	63 (98.4%)	48 (94.1%)	
**Complications**			
Intestinal obstruction			1.000
Yes	3 (4.7%)	2 (3.9%)	
No	61 (95.3%)	49 (96.1%)	
Intestinal perforation			1.000
Yes	1 (1.6%)	0	
No	63 (98.4%)	50 (100%)	
Gastrointestinal bleeding			0.380
Yes	4 (6.3%)	1 (2.0%)	
No	60 (93.7%)	50 (98.0%)	
Toxic megacolon			1.000
Yes	1 (1.6%)	0	
No	63 (98.4%)	51 (100%)	

^a^Chi-square test or Fisher’s exact test was used. In the non-CMV infection group, the patient numbers for hematochezia and intestinal perforation do not add up to the total due to missing data

With regard to laboratory tests, the rates of eosinopenia (0.2(0.1, 0.5) vs 0.8(0.4,2.5), P<0.001), hypoalbuminea (30.09 ± 5.10 vs. 34.67 ± 6.61, P<0.001), low IgA (1.91(1.21,2.29) vs 2.30(1.83, 2.82), *P* = 0.011), low IgM (0.52(0.40,0.89) vs. 0.89 (0.68, 1.86), P = 0.002), low IgG (8.9 (6.28, 11.79) vs. 12.34 (9.40, 16.20), *P* = 0.001), high-sensitivity C-reactive protein (hsCRP) (42.67 (17.16, 74.65) vs. 9.92 (3.68, 37.71), P = 0.001), and hyponatremia (136.41 ± 3.70 vs. 138.42 ± 2.98, P = 0.001) in the active CMV infection group were higher than those in the noninfection group. The laboratory data are shown in Table 3.

**Table 3:** Analysis of laboratory tests between the CMV infection and non-CMV infection groups

Variable	CMV infection (n = 64)	Non-CMV infection (n = 51)	P
ASCA^a^			
Positive	1 (4.3%)	2 (16.7%)	0.266
Negative	22 (95.7%)	10 (83.3%)	
ANCA^a^			0.568
Positive	19 (54.3%)	19 (59.4%)	
Negative	16 (45.7%)	13 (40.6%)	
WBC (10^9/L)	7.82 ± 3.04	7.70 ± 3.17	0.843
Neu (10^9/L)	5.18 (3.58,6.94)	4.55 (3.36,6.9)	0.494
Eos (10^8/L)	0.2 (0.1,0.5)	0.8 (0.4,2.5)	<0.001
LY (10^9/L)	1.67 (1.21,2.35)	1.68 (1.20,2.29)	0.968
Hb (g/L)	103.06 ± 23.59	111.53 ± 23.27	0.057
PLT (10^9/L)	293 (244,364)	294 (255,330)	0.802
Albumin (g/L)	30.09 ± 5.10	34.67 ± 6.61	<0.001
Serum potassium (mmol/L)	3.78 ± 0.36	3.73 ± 0.36	0.466
Serum sodium (mmol/L)	136.41 ± 3.70	138.42 ± 2.98	0.001
ALT (U/L)	15 (10,26.75)	10 (8,16.25)	0.008
AST (U/L)	15 (11,19.25)	14.6 (11,20)	0.933
GGT (U/L)	19 (15,38)	17.0 (11.2,25.5)	0.034
ALP (U/L	62 (51.75,71.25)	64 (54.5,87.5)	0.332
IgA (g/L)	1.91 (1.21,2.29)	2.30 (1.83,2.82)	0.011
IgM (g/L)	0.52 (0.40,0.89)	0.89 (0.68,1.86)	0.002
IgG (g/L)	8.9 (6.28,11.79)	12.34 (9.40,16.20)	0.001
C3 (g/L)	0.90 (0.71,1.03)	1.02 (0.76,1.25)	0.044
C4 (g/L)	0.21 (0.16,0.24)	0.23 (0.15,0.28)	0.283
ESR (mm/h)	36.0 (17.0,53.0)	27.0 (9.5,50.0)	0.119
hsCRP (mg/L)	42.67 (17.16,74.65)	9.92 (3.68,37.71)	<0.001

^a^Chi-square test or Fisher’s exact test was used. ^b^ data are expressed as the mean ± SD and were determined by a t test. & data are expressed by median and interquartile ranges and were determined by the Mann-Whitney U test. Patient numbers for the ASCA and ANCA tests do not add up to the total due to missing data

Note: *ASCA* anti-saccharomyces cerevisiae antibody, *ANCA* anti-neutrophil cytoplasmic antibodies, *ESR* erythrocyte sedimentation rate, *CRP* C response protein

### Comparison and analyses of disease patterns and drug administration conditions

Compared with the non-CMV group, the active CMV infection group exhibited significantly more cases of E3 lesions, initial onset type, and severe disease (determined clinically by the Truelove and Witts’ score and endoscopically by the Mayo endoscopic score as previously described). The number of patients who used immunosuppressive agents or a large dose of steroids was significantly higher in the active CMV infection group than in the non-CMV infection group.

However, the two groups did not present significant differences in the use of biological agents; history of diabetes mellitus, appendectomy, smoking, or drinking; and familiar history of IBD. These results are compiled in Table 4.

**Table 4:** Analyses of risk factors between CMV infection and non-CMV infection

Indicator	CMV infection (n = 64)	Non-CMV infection (n = 51)	P^a^
Disease extent			0.050
E1	1 (1.6%)	1 (2.0%)	
E2	10 (16.1%)	17 (34.0%)	
E3	51 (82.3%)	32 (64.0%)	
Disease severity			0.003
Mild	6 (9.7%)	8 (15.7%)	
Moderate	18 (29.0%)	28 (54.9%)	
Severe	38 (61.3%)	15 (29.4%)	
Disease type			0.008
Initial onset type	19 (30.6%)	5 (9.8%)	
Chronic recurrence type	43 (69.3%)	46 (90.2%)	
Immunosuppressive agents			0.042
Yes	12 (18.8%)	3 (5.9%)	
No	52 (81.2%)	48 (94.1%)	
steroid			<0.001
No	11 (17.2%)	33 (64.7%)	
Small dose	6 (9.4%)	12 (23.5%)	
Moderate dose	6 (9.4%)	2 (3.9%)	
Large dose	41 (64.1%)	4 (7.8%)	
IFX			0.092
Yes	4 (6.3%)	0	
No	60 (93.7%)	51 (100%)	
5-ASA			0.905
Yes	56 (87.5%)	45 (88.2%)	
No	8 (12.5%)	6 (11.8%)	

^a^Chi-square test or Fisher’s exact test was used. Some patient numbers do not add up to the total due to missing data

Note: *IFX* infliximab

### Multivariate analyses of independent risk factors for the development of active CMV infection in UC patients

As shown in Table 5, high-dose steroid treatment and immunosuppressive agents were risk factors for CMV infection; i.e., the risk of active CMV infection in patients who used glucocorticoids at large doses and immunosuppressive agents were 13.55 and 11.23 times higher, respectively, than those in patients who did not receive these treatments. Conversely, increases in eosinophil and albumin levels were protective factors against CMV infection. With every 0.1*10^9/L increase in the peripheral blood eosnophil level, the risk of CMV infection in UC patients decreased by 80.8% (1–0.192). With every 1 g/L increase in the serum albumin level, the risk of CMV infection in UC patients decreased by 16.1% (1–0.839). IgA, IgG, IgM, C3 and C4 were not included in the multivariate model due to missing data.

**Table 5:** Multivariate analyses

Variable	OR	95% CI	P
High-dose glucocorticoid	13.55	2.49–73.61	0.003
Immunosuppressive agent	11.23	1.05–119.99	0.045
Peripheral blood eosinophils	0.192	0.048–0.763	0.019
Serum albumin	0.839	0.728–0.967	0.015

## Discussion

The association between CMV infection and UC has received increasing attention in recent years. The results of our previous study [13] showed that the development of CMV colitis due to CMV infection causes colonoscopic changes in UC patients, presenting as punched-out and longitudinal ulcers. These changes draw increasing attention on the diagnosis and treatment of CMV infection and CMV colitis in active UC. Meanwhile, defective mucosal immunity, immunomodulator therapy and malnutrition contribute to the risk of developing CMV infection in patients with moderate-to-severe active UC. To better understand the influence and risk factors of CMV infection in active UC, we performed a multicenter case-control study to assess the clinical symptoms, complications, biochemical indicators and effect of therapeutic drugs on the development of infection in patients with patients with moderate-to-severe active UC. The results revealed prominent presentations of abdominal pain, fever, and oral ulcers after CMV infection, whereas the rates of gastrointestinal perforation, gastrointestinal bleeding, and toxic megacolon between the infection and noninfection groups did not appear significantly different. The probability of CMV infection induction increased with the use of glucocorticoids and immunosuppressive agents, whereas biological agents had no effect. Lower Ig levels and the initial onset type appeared more prominent in the CMV infection group than in the non-CMV infection group. Multivariate analysis showed that high-dose glucocorticoid treatment and immunosuppressive agents were independent risk factors for CMV infection, while low albumin and eosinophil levels were also risk factors for CMV infection in UC patients.

The major clinical manifestations of CMV infection are fatigue and fever. If the intestinal tract is involved, watery diarrhea, bloody stool, and abdominal pain might occur. This study analyzed the clinical characteristics of UC patients with and without CMV infection. The percentages of fever and abdominal pain in patients with CMV infection were significantly higher than those in patients without CMV infection, whereas no significant differences were observed in hematochezia and diarrhea incidence between the two groups. One possible reason for this result is that diarrhea and hematochezia are the major clinical manifestations for patients with active UC; therefore, significant differences between the two groups would be difficult to demonstrate.

With regard to complications, this study did not find significant differences in the proportions of complications between the CMV infection and noninfection groups. A recent study [14] reported that patients with CMV infection have a worse prognosis and are more prone to complications and increased colectomy rates than noninfected individuals. However, other studies did not support these results, possibly due to different inclusion criteria; for example, some studies enrolled patients with CMV infection, while others enrolled patients with CMV colitis. In addition, the results of this study indicated that patients with extensive colonic UC and severe UC are prone to CMV infection. These results were in accordance with several other studies [15, 16]. Recent basic research showed that CMV colitis was mediated by CMV-infected monocytes that recruit to the mucosa, not resident macrophages [17]. Hommes [18] proposed that the cytokine milieu of the colon, rich in TNFα and interferon-γ in active UC, leads to differentiation of CMV-infected monocytes into macrophages with reactivation of the latent disease. Therefore, the possibility of CMV infection should be considered for patients in the active stage with fever and abdominal pain that do not conform to the disease condition. In addition, the possibility of CMV infection should always be considered for patients with the E3 type and severe UC in the active stage.

The laboratory analysis results showed multiple abnormal indicators in the CMV infection UC group. The significant increase in hsCRP, an inflammatory indicator, suggests that patients with severe inflammation activity are more prone to CMV infection. This result was consistent with the aforementioned results showing severe UC patients to be prone to CMV infection. The hemoglobin level in the CMV infection group also was decreased compared with that in the noninfection group. Although a significant *P* value was not obtained (*P* = 0.054), these results nonetheless indicate that CMV infection can aggravate the condition of UC patients. Furthermore, serum sodium levels in the CMV infection group were decreased, and there are two possible explanations for this result. First, the high inflammatory activity aggravated the disease, and diarrhea caused by CMV infection resulted in hyponatremia. The other explanation addresses diarrhea resulting in hyponatremia, which might be associated with CMV infection. It has been shown that [19] hyponatremia will result in cellular immune dysfunction, and abnormal cellular immunity probably promotes CMV infection.

The results of this study showed significant reductions in immunoglobulin (IgG, IgM, and IgA) levels in the CMV infection group. Immunoglobulins (Ig) comprise a group of globulins with immune functions that can interact with specific antigens, and studies [20, 21] have shown that hypoimmunoglobulinemia is closely associated with refractory CMV disease. A reduction in Ig levels can increase susceptibility to CMV infection and promote the occurrence and aggravation of CMV infection. CMV infection also affects the stability of immune function and the balance of T cell subsets, and the presence of lymphokine-activated killer (LAK) cells will significantly decrease the activity of natural killer (NK) cells and aggravate reductions in Ig levels [22]. Studies [23, 24] in recent years have indicated that a reduction in serum Ig levels is common in adults and children with IBD, with one report [23] showing low IgG, IgG1, IgA, and IgM levels in 22.7, 23.4, 7.9, and 10.9% of IBD patients, respectively. IgAs, which are distributed on the surface of cells in the eyes and in the respiratory, gastrointestinal, and genitourinary tracts, are secretory Igs and the first-line defense against microorganisms. Although studies on the association between IgA and IBD are scarce, Zhou et al. [22] showed a significant reduction in serum IgA levels in patients infected with CMV in combination with other viruses. The results of this study suggest that attention should be paid to changes in Ig levels in research. Unfortunately, Ig was not included in the multivariate analysis due to missing data. Further expanding the sample size may help to obtain meaningful results.

Our study found increased rates of CMV infection in patients who received glucocorticoids and immunosuppressive agents. These results are similar to those of the majority of studies [25, 26]. In 2017, Shukla [27] et al. performed a meta-analysis and reported that exposure to corticosteroids (CS) (12 studies, 1180 patients, 52.3% exposed; OR, 2.05; 95% CI, 1.40–2.99) and thiopurines (14 studies, 1273 patients, 24.1% exposed; OR, 1.56; 95% CI, 1.01–2.39) was associated with increased risk of CMV activation. However, some of the studies included in this review did not clearly identified the severity of UC. Meanwhile, this meta-analysis did not evaluate the different influence of mild, moderate or large dose of glucocorticoids on CMV infection. Our results showed that patients who used large dose of steroids was significantly higher in the active CMV infection group than in the non-CMV infection group. No difference was found in mild or moderate dose of steroid. Therefore, the OR value of steroids and immunosuppressive agents in moderate to severe UC might be different to Tushar et al. reported. Besides, the retrospective case-control nature of this study might cause deviation in conclusion. Perspective cohort studies will be required for further investigation.

Similar to the results of another study [28], we did not find an increased risk of CMV infection due to the use of tumor necrosis factor (TNF) inhibitors because TNF inhibitors suppress TNF, an important cytokine that promotes CMV reactivation.

After removing confounding factors from the multivariate analyses, high-dose glucocorticoid treatment and immunosuppressive agents were shown to be independent risk factors for CMV infection in UC patients, increasing the risk of CMV infection in UC patients by 13.55-fold and 11.23-fold, respectively. Therefore, CMV infection should be strictly screened and monitored in these cases. Meanwhile, unnecessary long-term treatment with glucocorticoids at a high dose and immunosuppressive agents should be avoided.

Relatively few studies on the risk of CMV infection in UC patients with eosinopenia have been published. The eosinophil results are another highlight of our study, as the risk of CMV infection in UC patients was shown to decrease by 80.8% (1–0.192) with every 0.1*10^9/L increase in the peripheral blood eosnophil level. In general, more attention should be paid to an increased eosinophil level because this phenomenon can promote gastrointestinal inflammation and the release of cytokines, chemical factors, and lipid regulators. An increase in eosinophils is also associated with IBD and some gastrointestinal diseases. Studies [29] in recent years have shown that eosinophils, a component of the innate immune system, have the capacity to respond to pathogen-related molecules and are closely associated with infection, and a significant reduction in peripheral blood eosinophil numbers usually occurs during the clearance of infection. In addition, eosinopenia is associated with acute infection [30], and studies [31, 32] have also shown that a reduction in eosinophils is a sensitive and reliable indicator for distinguishing between infection- and noninfection-related sepsis in intensive care units (ICUs).

The multivariate analyses suggested the following: long-term treatment with glucocorticoids at a high dose and immunosuppressive agents should be avoided; patients’ normal immune statuses should be maintained; Ig treatment should be properly applied for severe infections; and UC patients with low eosinophil levels should be monitored for CMV infection. Furthermore, patients with CMV infection showed lower serum albumin levels, which was probably due to sever inflammatory process (present as high hsCRP levels) and insufficient intake. A few studies [31, 33, 34] have indicated that albumin is a nutritional and inflammatory marker that reflects the potential for infection or disease activity. However, the effect of treatment targeting at increasing albumin in CMV infection has not been fully illustrate. Further studies are needed to investigate the role of albumin intervention in CMV infection in UC patients.

The limitation of this study is that due to its case-control nature, whether the indicators are the causes or results of CMV infection cannot be determined. For example, we found CMV group to have significantly higher use of high dose steroids, eosinopenia, elevated CRP. However, explanation remains that patients with more severe UC, which are more likely to be on high dose steroids, which ultimately are the patients with higher seropositivity for CMV. Although multivariate analysis showed that high dose steroids and eosinopenia are both independent risk factors for CMV infection in active UC, cohort studies will be required for further confirm and investigation. Moreover, this study discussed CMV infection in active UC but did not detailly discussed CMV colitis. Further studies are needed to specifically investigate the risk factors of CMV colitis in active UC. Meanwhile, other aspects (like genetics, hormonal changes and stress) which could contribute to CMV infection were not identified in this study. The influence of these aspects remains to be discussed in further studies. Besides, due to missing data, immunoglobulin was not included in the multivariate analysis. Further prospective studies are needed to better explore the role of immunoglobulin and compliment in CMV infection in active UC.

In summary, CMV infection may occur at the active stage of UC due to changes in the immune status or disease condition; in turn, CMV infection aggravates the condition of UC patients, making the inflammatory disease difficult to control. For patients with extensive UC, treating inflammation, correcting anemia and hyponatremia, improving immunoglobulin function and monitoring low serum and eosinopenia levels may help prevent CMV infection and lead to a better prognosis. Furthermore, the molecular mechanisms underlying the association between Ig, eosinopenia and UC combined with CMV infection are worthy of further study.

## Supplementary information

**Additional file 1 Supplementary Table 1** Clinical Manifestations of Patients Satisfying Different Diagnostic Criteria.

## Data Availability

The datasets used and/or analyzed during the current study available from the corresponding author on reasonable request.

## Abbreviations

CMV: Cytomegalovirus
UC: ulcerative colitis
TNF: Tumor necrosis factor
ICUs: Intensive care units
ECCO: The European Crohn’s and Colitis Organization
CMV pp65: HCMV pp65 antigen
qPCR: quantitative polymerase chain reaction
LAK: Lymphokine-activated killer
NK: Natural killer
Ig: Immunoglobulins
hsCRP: High-sensitivity C-reactive protein
