# Supplementary material for: IgA, albumin, and eosinopenia as early indicators of cytomegalovirus infection in patients with acute ulcerative colitis
Source: BMC Gastroenterol. 2020 Sep 5;20:294. doi: 10.1186/s12876-020-01434-5 (PMC7487863; doi:10.1186/s12876-020-01434-5)
Supplement: Supplementary file 1 — Additional file 1 Supplementary Table 1 Clinical Manifestations of Patients Satisfying Different Diagnostic Criteria. [file 12876_2020_1434_MOESM1_ESM.docx]

**Supplementary Table 1 Clinical Manifestations of Patients Satisfying Different Diagnostic Criteria**

|  | CMV-DNA>500 copies/ml | CMV-IgM Positive | CMV-pp65 Positive | CMV Colitis |
| --- | --- | --- | --- | --- |
|  | N=56 | N=13 | N=24 | N=39 |
| Male | 36 (64.3%) | 9 (69.2%) | 15 (62.5%) | 25 (64.1%) |
| Age at onset | 44.8±13.3 | 45.6±14.7 | 46.5±14.0 | 45.2±13.2 |
| Fever | 31 (55.4%) | 6 (46.2%) | 11 (45.8%) | 20 (51.3%) |
| Abdominal pain | 45 (80.4%) | 10 (76.9%) | 18 (75.0%) | 30 (76.9%) |
| Eosinophil (10^8/L) | 0.7±1.4 | 0.8±2.0 | 0.8±1.9 | 0.8±0.2 |
| Albumin (g/L) | 29.6±5.0 | 29.7±4.8 | 29.5±4.0 | 29.6±4.6 |
| ALT (g/L) | 23.5±24.0 | 23.7±14.3 | 24.8±27.8 | 25.9±27.8 |
| hsCRP (mg/L) | 58.3±65.8 | 55.9±61.2 | 50.5±33.8 | 64.1±51.5 |
| Disease extent: E3 | 47 (83.9%) | 11 (84.6%) | 21 (87.5%) | 35 (89.7%) |
| Disease severity: severe | 38 (67.8%) | 8 (61.5%) | 16 (66.7%) | 30 (76.9%) |
| Large dose steroid | 39 (69.6%) | 8 (61.5%) | 16 (66.7%) | 25 (64.1%) |
| Immunosuppressive agents | 11 (19.6%) | 3 (23.1%) | 5 (20.8%) | 7 (17.9%) |
| Infliximab | 4 (7.1%) | 1 (7.7%) | 3 (12.5%) | 3 (7.7%) |
